# Developing a conceptual model of symptoms and impacts in progressive fibrosing interstitial lung disease to evaluate patient-reported outcome measures

**DOI:** 10.1183/23120541.00681-2021

**Published:** 2022-05-03

**Authors:** Marlies Wijsenbeek, Maria Molina-Molina, Olivier Chassany, John Fox, Liam Galvin, Klaus Geissler, Katherine M. Hammitt, Michael Kreuter, Teng Moua, Emily C. O'Brien, Ashley F. Slagle, Anna Krasnow, Matthew Reaney, Michael Baldwin, Natalia Male, Klaus B. Rohr, Jeff Swigris, Katerina Antoniou

**Affiliations:** 1 Dept of Respiratory Medicine, Erasmus University Medical Centre, Rotterdam, The Netherlands; 2 Dept of Pneumology, Unit of Interstitial Lung Diseases, University Hospital of Bellvitge, Institute for Biomedical Research (IDIBELL), Barcelona, Spain; 3 Patient-Reported Outcomes Research Unit, Université de Paris, Paris, France; 4 Health Economics Clinical Trial Unit (URC-ECO), Hotel-Dieu Hospital, AP-HP, Paris, France; 5 Foxworthy Healthcare Consulting, Ada, MI, USA; 6 European Idiopathic Pulmonary Fibrosis and Related Disorders Federation, Overijse, Belgium; 7 Patient Support Group, Lungenfibrose eV, Essen, Germany; 8 Sjögren's Foundation, Reston, VA, USA; 9 Center for Interstitial and Rare Lung Diseases, Department of Pneumology, Thoraxklinik, University of Heidelberg, Member of the German Center for Lung Research, Heidelberg, Germany; 10 Division of Pulmonary and Critical Care Medicine, Mayo Clinic, Rochester, MN, USA; 11 Duke Clinical Research Institute, Durham, NC, USA; 12 Aspen Consulting, LLC, Steamboat Springs, CO, USA; 13 Patient Centred Solutions, IQVIA, London, UK; 14 Patient Centred Solutions, IQVIA, Reading, UK; 15 Boehringer Ingelheim International GmbH, Ingelheim am Rhein, Germany; 16 National Jewish Health, Denver, CO, USA; 17 Laboratory of Cellular and Molecular Pneumonology, Dept of Respiratory Medicine, School of Medicine, University of Crete, Crete, Greece

## Abstract

**Background:**

An understanding of the experience of patients with progressive fibrosing interstitial lung disease (PF-ILD) is needed to select appropriate patient-reported outcome measures (PROMs) to evaluate treatment effect in clinical trials.

**Methods:**

A systematic literature review was conducted to develop a preliminary conceptual model of the symptoms experienced by patients with PF-ILD and the impacts the disease has on them. An online survey and consensus meetings were then conducted with 12–14 stakeholders (patients, clinicians, regulatory and payer advisors) to refine the conceptual model and critically appraise how key concepts should be measured by PROMs. PROMs assessed included Living with Idiopathic Pulmonary Fibrosis, Living with Pulmonary Fibrosis, the King's Brief Interstitial Lung Disease questionnaire, Cough and Sputum Assessment Questionnaire, Evaluating Respiratory Symptoms, Leicester Cough Questionnaire, Functional Assessment of Chronic Illness Therapy (Dyspnoea/Fatigue) and St George's Respiratory Questionnaire for Idiopathic Pulmonary Fibrosis.

**Results:**

The literature review identified 36 signs/symptoms and 43 impacts directly or indirectly related to pulmonary aspects of PF-ILD. The most relevant symptoms identified by participants included shortness of breath on exertion, fatigue and cough; relevant impacts included effects on physical functioning, activities of daily living and emotional wellbeing. These are presented in a conceptual model. Consensus opinion was that existing PROMs need further modification and validation before use in clinical trials.

**Conclusions:**

The conceptual model improves understanding of the symptoms and impacts that living with PF-ILD has on patients’ wellbeing. It can help to inform the choice of PROMs in clinical trials and highlight aspects to assess in the clinical care of patients with PF-ILD.

## Abstract 2

**This conceptual model of symptoms and impacts in progressive fibrosing interstitial lung disease can help guide patient-reported outcome measure selection in clinical trials** https://bit.ly/3tReuGi

## Introduction

Interstitial lung diseases (ILDs) are a heterogeneous group of lung disorders, characterised by inflammation and scarring of the lung tissue [1]. Fibrotic ILDs occur in a range of disorders, including connective tissue diseases (*e.g.* systemic sclerosis, Sjögren disease, rheumatoid arthritis), hypersensitivity pneumonitis or with no obvious underlying cause [1–3]. Idiopathic pulmonary fibrosis (IPF) is the archetypal fibrotic ILD and is always progressive in the long term; its prevalence is estimated at eight to 60 cases per 100 000 people [1]. A proportion of patients with fibrotic ILDs other than IPF can develop a progressive fibrosing phenotype, with prevalence estimates of 2.2–20.0 per 100 000 people in Europe and 28.0 per 100 000 in the United States [4]. Regardless of aetiology, ILDs with a progressive phenotype display similar disease behaviour and are collectively referred to as progressive fibrosing ILDs (PF-ILDs), characterised by declining lung function, worsening symptoms and early mortality [2].

Symptoms associated with PF-ILD include shortness of breath, fatigue and cough [5, 6]. These can affect how patients feel and function in their daily lives, limit mobility and impair social participation and independence [7]. The emotional burden (anxiety, fear and depression) of PF-ILD has been linked to worsening symptoms and the weight of living with a progressive condition [8, 9]. Previous studies have examined how symptoms and impacts of PF-ILD affect health-related quality of life (HRQoL) [10], but none have comprehensively investigated what should be measured to fully assess the burden of PF-ILD or how it should best be captured to generate reliable and interpretable data that inform clinical decision-making.

As a first step, an understanding of which symptoms and impacts are most meaningful to patients is needed. Such information is key to determining how therapeutic strategies affect patients’ disease experience. Relevant perspectives on defining treatment benefit include those from people living with the disease (patients), treating the disease (clinicians) and making decisions about the (comparative) benefit–risks of different treatments (regulatory/payer advisors). Their perspectives can be integrated into a conceptual model that ultimately informs the assessment of treatment benefit that will be informative to patients, clinicians and other stakeholders [11].

Given their subjective nature, symptoms and impacts of disease are best captured directly through patient-reported outcome measures (PROMs). Regulatory bodies and patient advocacy organisations have emphasised the importance of PROMs in clinical trials to measure the efficacy of pharmacological interventions [12, 13]. In diseases such as pulmonary arterial hypertension and COPD, HRQoL is considered a key component of the end-point model by the European Medicines Agency [14, 15]. However, there are limited data in PF-ILD that support the use of any PROM for longitudinal research and clinical trials [10]. For use in this context, PROMs should be a comprehensive evaluation of the concepts of interest, easily understood and psychometrically sound [12]. Unfortunately, the PROMs used in PF-ILD research do not meet all these criteria, with the majority developed in patients with other respiratory diseases and adapted for PF-ILDs [10]. ILD-specific PROMs, such as the Living with Pulmonary Fibrosis (L-PF) [16] questionnaire and the King's Brief Interstitial Lung Disease (K-BILD) [17] questionnaire have not yet been comprehensively tested for reliability or validity in PF-ILD.

The first aim of this study was to use a systematic literature review, online survey and consensus meetings to develop and refine a conceptual model of signs/symptoms and impacts directly or indirectly related to pulmonary aspects of PF-ILD. This conceptual model will outline what should be measured to assess the burden of PF-ILD. The second aim was to critically appraise existing PROMs and seek stakeholder perspectives on how to measure the most relevant concepts defined by the conceptual model.

## Methods

The conceptual model of PF-ILD was developed in three steps: a systematic literature review, a stakeholder survey and consensus panel meetings. Stakeholders from Europe and the USA were invited to join the panel to participate in the online survey and consensus meetings. The clinicians all had expertise in treating patients with ILD or conducting PROMs research, and patient representatives were key members or leaders of IPF, ILD or connective tissue disease patient groups who had experience of living with PF-ILD. Payer and regulatory advisors all had expertise in clinical trial design and research.

### Aim 1: what to measure; developing a conceptual model of PF-ILD

#### Systematic literature review

A systematic literature review was conducted in electronic medical databases (Cochrane Library, Embase and MEDLINE/MEDLINE in Process) and supplemented with searches in patient blogs. The search strategy for the systematic literature review is outlined in supplementary figure S1; search terms are shown in supplementary tables S1 and S2. The search was conducted on 23 March 2020 and was limited to English-language publications within the previous 10 years. The focus of the search was to identify signs/symptoms and impacts relevant to understanding patients’ experiences of PF-ILD and its treatment, and excluded any that were unrelated to pulmonary aspects of PF-ILD or were unique to a single ILD type. The results from the systematic literature review were used to develop a preliminary conceptual model.

#### Online survey

All participants in the panel were sent an online survey and presented with a list of symptoms and impacts based on the preliminary conceptual model to further prioritise the concepts. They were offered an opportunity to add any concepts that they felt were missing from the preliminary conceptual model. Surveys were similar for each of the four types of respondents, with minor adaptations to maintain relevance and generate focused perspectives. For example, clinicians were asked to select the symptoms that indicated disease progression and the impacts of PF-ILD that their patients expressed as most troublesome. Patient representatives were asked which symptoms and impacts were the most disturbing/bothersome and the most important to target to improve their condition. Regulatory and payer advisors were asked to select the most important symptoms and impacts to include as part of a PROM evidence package in a submission for regulatory approval or coverage/reimbursement decision.

#### Consensus meeting and online poll

**FIGURE 1:**
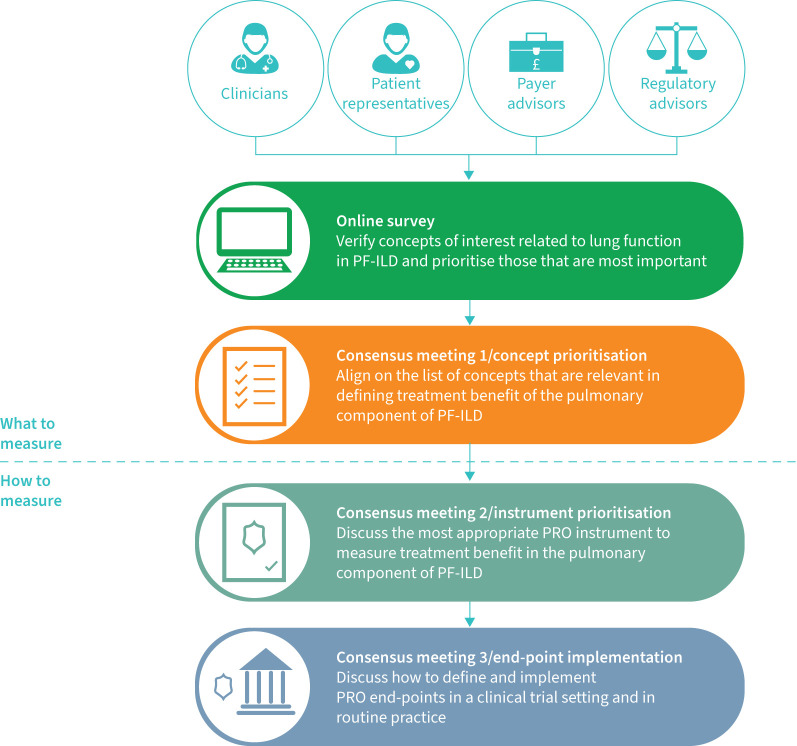
Flow of consensus meetings involving stakeholders. PF-ILD: progressive fibrosing interstitial lung disease; PRO: patient-reported outcome.

Three meetings were conducted with the stakeholders to develop consensus (figure 1). The first meeting focused on the conceptual model. The concepts generated from the systematic literature review and online survey were discussed and voted on. Stakeholders were asked in a poll to distribute 100 points across each of the symptoms and impacts based on how important measuring the concept was for determining treatment benefit. A separate poll was conducted to identify which characteristic (severity, frequency or interference with daily life) was considered the most important to measure for each of the prioritised concepts. Results were used as a basis for further discussions. The conceptual model was finalised after this consensus meeting, and a priority list of concepts was developed.

### Aim 2: how to measure; critical appraisal of PROMs in PF-ILD

#### PROM literature review and evaluation

PROMs previously used to measure HRQoL in ILD populations were identified through the initial systematic literature review and a targeted review of online databases (Patient-Reported Outcome and Quality of Life Instruments Database; ClinicalTrials.gov), and evaluated according to industry best-practice criteria [12]. PROMs with suitable content validity and psychometric properties were then evaluated through conceptual mapping with the final conceptual model (as developed in aim 1).

#### Consensus meetings

Two additional consensus meetings were held with the stakeholders. In the first, participants reviewed the evidence for PROMs and were asked to select the recall period, response scale and mode of administration that was most appropriate to measure the prioritised concepts. In the final meeting, participants discussed the key considerations for implementing PROM end-point assessment in clinical trials and clinical practice to evaluate treatment benefit.

## Results

### Data sources and participants

The systematic literature review identified 23 publications, 14 of which were qualitative studies (interviews or focus groups involving patients or patients and caregivers), with the remaining featuring registry data, surveys or questionnaires. Additionally, 10 blogs describing patient experiences were identified. 12 participants took part in the online survey, which included patient representatives (n=3), clinicians (n=5), regulatory advisors (n=2) and payer advisors (n=2). There were 14 participants in the consensus meetings (participants from the online survey plus two other clinicians).

### Development of the conceptual model

#### Systematic literature review

The publications and patient blogs identified 40 signs/symptoms and 48 impacts experienced by patients with PF-ILD. Four symptoms were excluded because they were unrelated to the pulmonary component of the PF-ILD or its treatment. Four impacts were excluded because they were not related to treatment or disease, and one impact was excluded because it was considered unique to a single type of PF-ILD. The remaining signs/symptoms (n=36) and impacts (n=43) were presented in a preliminary conceptual model.

#### Online survey

No new sign, symptom or impact concepts were raised by stakeholders in the online survey that were not already captured in the preliminary conceptual model. 10 signs/symptoms and 12 impacts from the preliminary conceptual model were prioritised by participants in the online survey (supplementary table S3), which were put forward to the consensus meeting.

#### First consensus meeting and online poll

**FIGURE 2:**
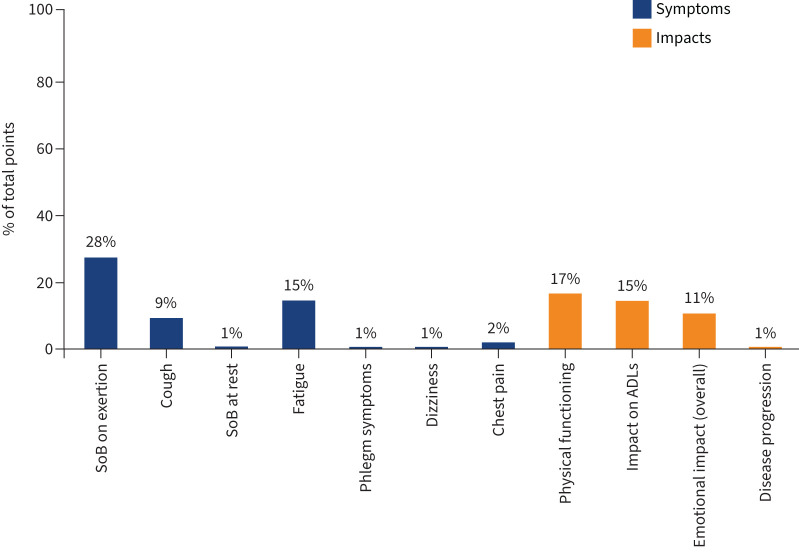
Important symptoms and impacts reported by participants in the online poll for determining treatment benefit. Other symptoms: chest discomfort 0%, other chest symptoms 0%, runny nose 0%. Other impacts: fear of infection 0%, loss of freedom 0%, oxygen use impacting on activities of daily living (ADLs) 0%, concern for family 0%, anxiety 0%, depression 0%, thoughts of death 0%, implication of disease 0%. SoB: shortness of breath.

The conceptual model was discussed at the first consensus meeting. One additional sign (muscle wasting) was raised by a patient representative and added to the final conceptual model. The results from the poll of the most relevant concepts to be measured for determining treatment benefit show that only seven signs/symptoms and four impacts were allocated points (figure 2).

**FIGURE 3:**
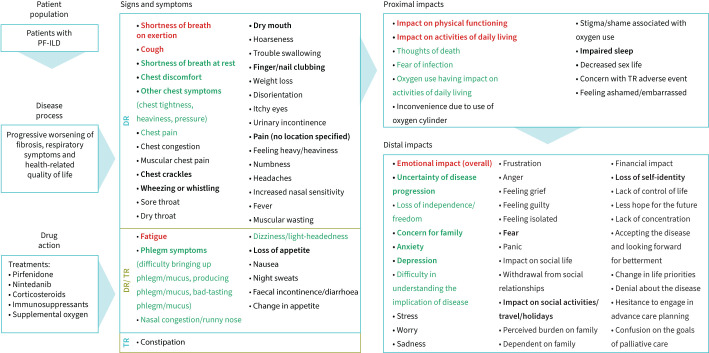
Final conceptual model. Concepts prioritised in the consensus meeting are in red and those prioritised in the online survey are in green. All concepts of high prevalence (at least one literature publication reporting a prevalence of ≥50%) are in bold. Impacts where the relationship to signs and symptoms was indirect/unclear are categorised as distal. PF-ILD: progressive fibrosing interstitial lung disease; DR: disease-related; TR: treatment-related.

**FIGURE 4:**
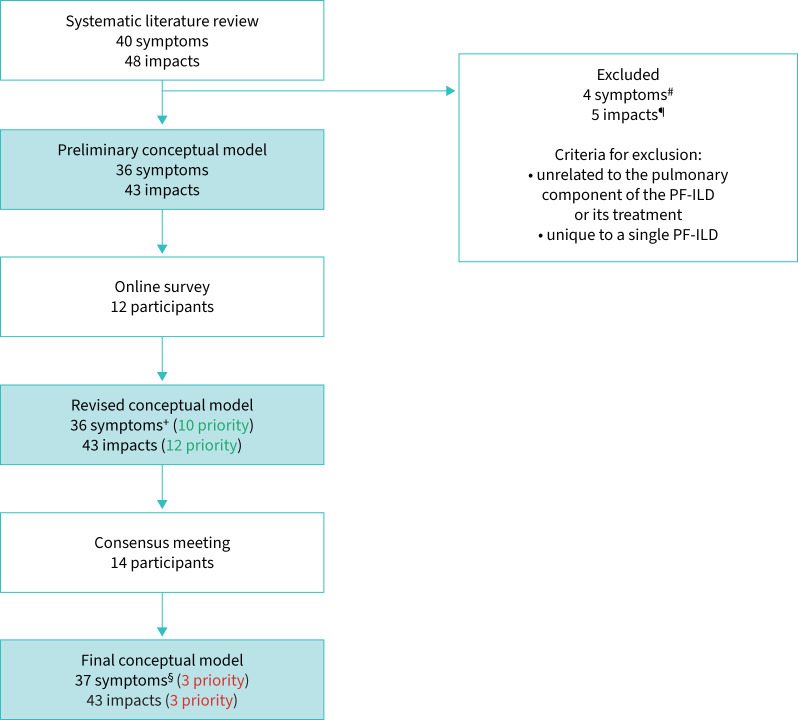
Process of developing the conceptual model. Green: prioritisation at online survey; red: prioritisation at consensus meeting. PF-ILD: progressive fibrosing interstitial lung disease. ^#^: muscle loss, stiffness in joints, joint pain and swollen and inflamed joints. ^¶^: lack of psychological support, lack of satisfaction with healthcare and positive feelings/experiences with healthcare, concerns with diagnosis and need for more disease awareness, being more vigilant towards avoiding antigens. ^+^: symptoms from the online survey were adapted from the preliminary consensus model: three phlegm symptoms (difficulty bringing up phlegm, producing phlegm and bad-tasting phlegm) were grouped together; the shortness of breath (SoB) concept was split into SoB at rest and SoB on exertion; change in appetite added to differentiate from loss of appetite. ^§^: muscular wasting was added as part of the final conceptual model.

Of the seven signs/symptoms, meeting participants agreed that the signs/symptoms that best characterise PF-ILD are shortness of breath on exertion, cough and fatigue. The impacts that were considered as most relevant out of the four with allocated points were physical functioning, impact on activities of daily living (ADLs) and emotional impacts. The final conceptual model is shown in figure 3. The signs/symptoms are presented in disease- and/or treatment-related categories. Impacts were categorised as proximal (direct) or distal (indirect). Figure 4 shows the process of developing the conceptual model.

**FIGURE 5:**
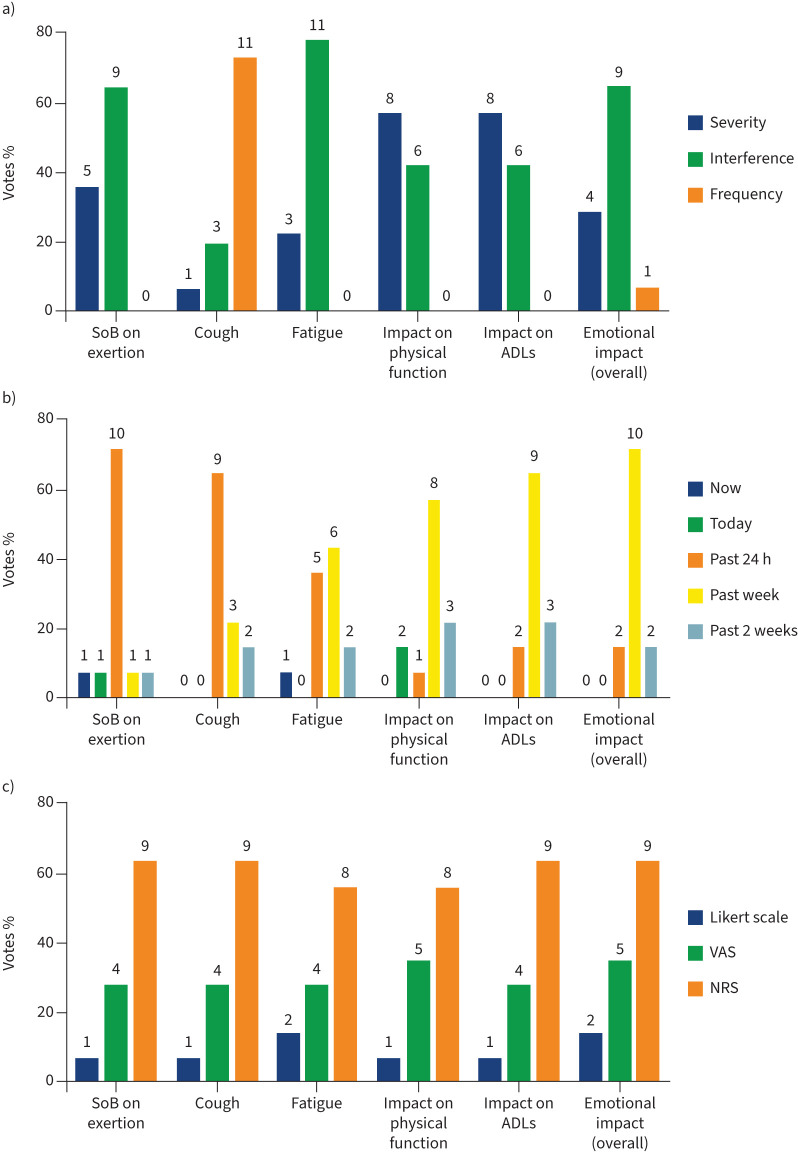
a) The most important characteristics of the prioritised concepts; b) the most appropriate recall period; c) the most appropriate response scale reported by participants in the meeting to measure prioritised concepts. The total number of votes was 14 for most concepts, except where participants selected two categories when voting. Data are presented as percentage of total votes, with the actual number of votes shown above each bar. SoB: shortness of breath; ADLs: activities of daily living; VAS: visual analogue scale; NRS: numeric rating scale.

A second poll identified the most important characteristic (severity, frequency or interference with daily life) to measure for each of these three prioritised symptoms and three prioritised impacts. Interference with daily life was the most selected aspect to measure for shortness of breath on exertion, fatigue and emotional wellbeing. Frequency was most selected for cough (figure 5a). Severity was the most selected characteristic to measure for impact on physical functioning and ADL.

### Measuring concepts of interest

**FIGURE 6:**
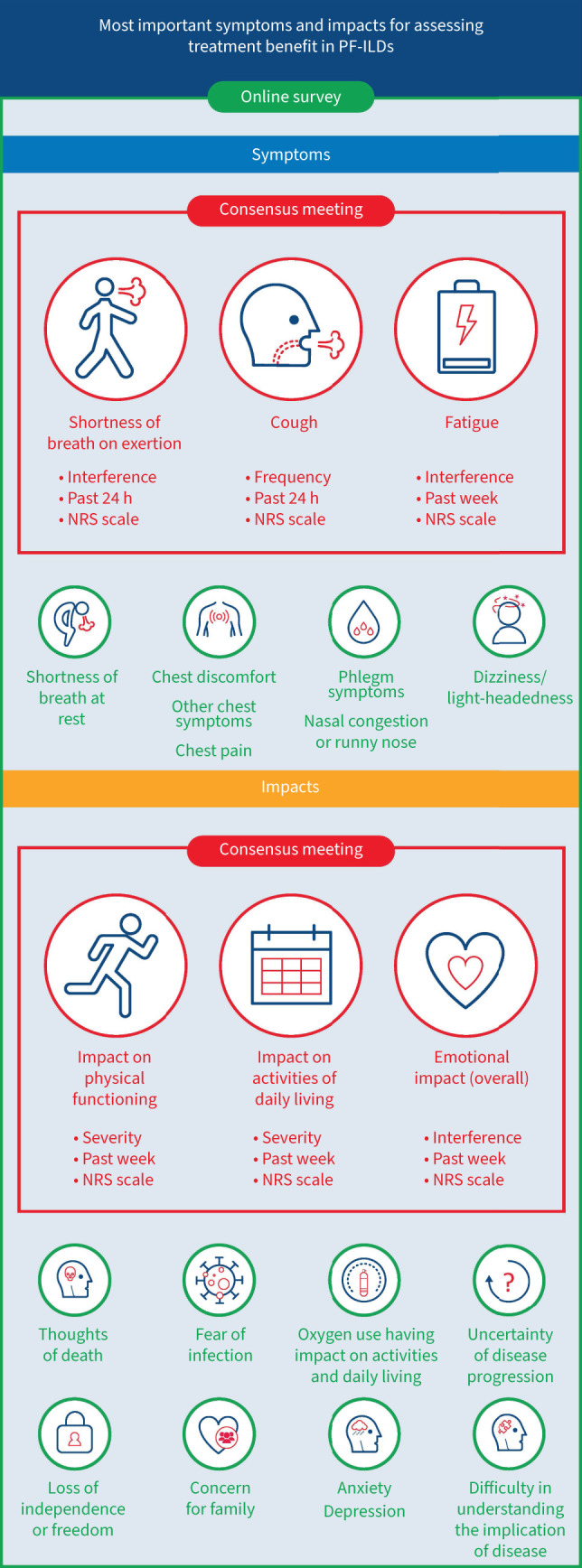
Priority concepts for defining treatment benefit in progressive fibrosing interstitial lung disease (PF-ILD). NRS: numeric rating scale.

It was agreed by all stakeholders that prioritised concepts are best measured using PROMs. Participants discussed appropriate recall periods and response scales for the collection of PROM data. The participants selected a recall period of 24 h as most relevant for shortness of breath on exertion (10 out of 14 participants) and cough (nine out of 14 participants). For fatigue, six out of 14 participants selected a recall period of 1 week and five out of 14 selected a period of 24 h. A recall period of 1 week was most selected for all the impacts, and all participants suggested that they would be able to recall and answer questions related to the past week (figure 5b). Although patient representatives indicated a preference for visual analogue scales, a numeric rating scale was better accepted by payer and regulatory advisors and given the most votes (figure 5c). There was consensus that an electronic version of a questionnaire was the most appropriate option (10 out of 13) for administration. Our results on priority concepts and how to measure them are summarised in figure 6.

Nine existing PROMs were critically appraised against the conceptual model, and specifically against the prioritised concepts to evaluate if they appropriately measured the three prioritised signs/symptoms and three prioritised impacts in line with the proposed characteristics (severity, frequency or interference with daily life). The recall period and response scale(s) of the PROMs were evaluated for consistency with the proposed approach suggested by stakeholders (table 1).

**TABLE 1:** Summary of patient-reported outcome measure (PROM) questionnaires and concepts captured

	**Content validity**	**Psychometric properties**	**Items**	**Recall period**	**Response scale**	**SoB on exertion**	**Cough**	**Fatigue**	**Physical functioning**	**ADL**	**Emotional wellbeing**
**K-BILD [** **17, 18** **]**	Input from patients with ILD (including IPF)	Cross-sectional and longitudinal measurement properties established in patients with IPF	15	2 weeks	7-point Likert scale	F: 3 items			INF: 1 item	INF: 1 item	
**SGRQ-I [** **18, 19** **]**	Target: IPF Rasch analysis using HRQoL data from RCT	Cross-sectional and/or without longitudinal measurement properties established in patients with IPF	34	Current/3 months/6 months/12 months	3–5-point Likert scale	S: 1 item; INC: 4 items	S: 2 items; INC: 1 item	INC: 1 item	S: 8 items; INC: 1 item; INF: 2 items	INC: 5 items; INF: 3 items	
**L-IPF [** **20** **]**	Input from patients with IPF	Cross-sectional measurement properties established patients with IPF	35	24 h (symptoms) 1 week (impacts)	0–4 NRS format	S: 7 items; INC: 1 item; INF: 3 items	F: 3 items; S: 2 items; INF: 1 item	S: 3 items; INF: 1 item	S: 6 items; INF: 1 item	S: 5 items; INF: 2 items	
**L-PF [16]**	Input from patients with PF-ILD	Not available	44	24 h (symptoms) 1 week (impacts)	0–4 NRS format	S: 12 items; F: 1 item; INF: 3 items; INC: 1 item	F: 5 items; S: 2 items; INC: 1 item	S: 3 items; INF: 1 item	S: 8 items INF: 1 item	S: 7 items; INF: 3 items	
**CASA-Q [** **21, 22** **]**	Input from patients with chronic bronchitis	Cross-sectional and longitudinal measurement properties established in patients with COPD and chronic bronchitis	20	1 week	5-point Likert scale	F: 2 items	S: 1 item; F: 2 items	F: 1 item	INF: 2 items	INF: 4 items	
**LCQ [** **23** **]**	Input from patients with chronic cough	Cross-sectional and longitudinal measurement properties established in patients with chronic cough or COPD	19	2 weeks	7-point Likert scale		S: 1 item; F: 1 item	F: 2 items		INF: 2 items	
**E-RS (COPD+IPF) [** **24, 25** **]**	Input from patients with COPD	Cross-sectional and longitudinal measurement properties established in patients with COPD	11	24 h	5-point Likert scale	S: 5 items	F: 1 item	S: 1 item	INF: 1 item	INF: 2 items	
	Input from patients with IPF	Cross-sectional and longitudinal measurement properties established in patients with IPF									
**FACIT-Dyspnoea [** **26** **]**	Input from patients with dyspnoea	Cross-sectional measurement properties established in patients with dyspnoea	Item bank: 33; short form: 10	1 week	4-point Likert scale	S: 34 items			INF: 12 items	INF: 18 items	
**FACIT-Fatigue [** **27** **]**	Input from patients with anaemic cancer	Cross-sectional measurement properties established in a mixed cancer patient population	40	1 week	5-point Likert scale			S: 7 items INF: 1 item	INF: 3 items S: 2 items	INF: 1 item	

SoB: shortness of breath; ADL: activity of daily living; K-BILD: King’s Brief Interstitial Lung Disease; SGRQ-I: idiopathic pulmonary fibrosis (IPF)-specific version of St George's Respiratory Questionnaire; L-IPF: Living with Idiopathic Pulmonary Fibrosis; L-PF: Living with Pulmonary Fibrosis; CASA-Q: Cough and Sputum Assessment Questionnaire; LCQ: Leicester Cough Questionnaire; E-RS: Evaluating Respiratory Symptoms; FACIT: Functional Assessment of Chronic Illness Therapy; ILD: interstitial lung disease; F: frequency; INF: interference; HRQoL: health-related quality of life; RCT: randomised controlled trial; PF-ILD: progressive fibrosing interstitial lung disease; S: severity; INC: incidence; NRS: numeric rating scale.

Only the L-PF and the K-BILD were developed in patients with ILD, with the L-PF specifically targeting the PF-ILD population [16]. Given this, the use of a single PROM that measured all prioritised concepts *versus* a “combination approach” (using two or more existing PROMS that were concept-specific) was discussed. Approximately half of the participants preferred a single instrument, specific to PF-ILD, that measures all priority concepts, whereas half preferred a combination of concept-specific instruments.

No consensus was reached on the ideal PROM for PF-ILD, but the L-PF questionnaire met several, though not all, expectations (table 2). The L-PF is longer than the other questionnaires (44 items) and may not be sufficiently granular for measuring fatigue (only one impact item measuring fatigue). It was suggested that a generic questionnaire such as the EuroQoL five-dimension questionnaire should be considered for utility analyses to allow comparison with other studies. A fatigue-specific PROM (*e.g.* Functional Assessment of Chronic Illness Therapy – Fatigue) could be used to measure fatigue in clinical trials if a treatment benefit on fatigue is expected.

**TABLE 2:** Review of the Living with Pulmonary Fibrosis (L-PF) questionnaire for measuring symptoms and impacts of progressive fibrosing interstitial lung disease (PF-ILD)

**Consensus recommendation**	**Relevant items from the L-PF**	**Comments**
**Interference on daily life of SoB (on exertion) in past 24 h**	Interference on daily life of SoB Impacts module: 1) How much did shortness of breath prevent you from doing things you wanted to do? 2) How much did fear of becoming too short of breath limit your physical exertion? 17) How has shortness of breath affected your quality of life?	The L-PF has 17 items measuring the incidence, severity, frequency and interference of SoB upon exertion. Further thought is needed as to whether the three impact items on interference should be used in isolation and how a score would be calculated. The recall period for the three items on interference also assesses the past week, rather than 24 h. The other 14 items in the symptoms module assesses SoB over the past 24 h. The L-PF response scale (0–4 numeric scale) is consistent with consensus recommendations
**Frequency of cough in past 24 h**	Frequency of cough Symptoms module: 13) Over the last 24 h, how often did you cough? 14) Over the last 24 h, how often did you cough when you took a deep breath? 15) Over the last 24 h, how often did you cough when you were breathing hard or fast? 16) Over the last 24 h, how often did you cough when you over-exerted yourself? 17) Over the last 24 h, how often did coughing make you short of breath?	The L-PF has five items for measuring the frequency of cough over the past 24 h. Three other items assess the severity and incidence of cough.
**Interference on daily life of fatigue in past week**	Interference on daily life of fatigue Impacts module: 19) How much has your energy level affected your quality of life?	The L-PF questionnaire may lack specificity in the impact items for fatigue. There is only one fatigue-related item, which focuses on energy levels. If a treatment benefit on fatigue was expected, a fatigue-specific questionnaire may be needed in addition to the L-PF
**Severity of physical functioning limitations in past week**	Severity of physical functioning limitations Symptoms module: 4) How short of breath did walking up a short, gradual incline make you? 6) How short of breath did walking outside on a level surface make you? 7) How short of breath did walking from room to room inside your home make you? 8) How short of breath did getting ready to leave your home (*e.g.* find keys, put on coat, lock doors) make you? 10) How short of breath did doing light cleaning around the house make you? 12) How short of breath did lifting and carrying a light load a short distance make you? Impacts module: 14) How much did you have to pace yourself to make it through the day? 15) How much time did it take to get yourself ready to leave the house?	The L-PF has six items in the symptoms module (24 h) and two items in the impacts module (past week) that assess the severity of physical functioning. The items in the symptoms module focus on SoB.
**Severity of ADL limitation in past week**	Severity of ADL limitation Symptoms module: 1) How short of breath did getting dressed make you? 2) How short of breath did walking up one flight of stairs make you? 3) Over the last 24 h, how short of breath have you been while sitting down, relaxing, reading or watching TV? 5) How short of breath did grooming make you? 6) How short of breath did walking outside on a level surface make you? 9) How short of breath did bathing or showering make you? Impacts module: 3) How was your stamina when you exerted physically?	The L-PF has six items in the symptoms module (24 h) and one item in the impacts module (past week) that assess the severity of ADL limitation. The items in the symptoms module focus on SoB.
**Interference on daily life of emotional wellbeing in past week**	NA	NA

SoB: shortness of breath; ADL: activity of daily living; NA: not applicable.

### Outcome and end-point measurement in clinical trials and clinical practice

When discussing the end-point model of PROMs, all participants agreed that improvement was the ideal outcome for treatments, but would accept maintenance or prolonging time to deterioration if relevant. When asked what type of end-point would be most relevant, regulatory advisors preferred a comprehensive analysis of end-points (change from baseline, time to event, and responder analyses), whereas patient representatives and clinicians preferred change from baseline. Payer and regulatory advisors both considered meaningful change thresholds for end-points to be important for evaluating treatment. When discussing end-point positioning, payer and regulatory advisors expected PROMs to be secondary end-points or a co-primary end-point alongside an objective measure (*e.g.* forced vital capacity).

## Discussion

We developed a conceptual model to better understand the patient experience of PF-ILD. Results from this study provide consensus, from the perspective of multiple stakeholders, on the key symptoms and impacts that should be assessed to determine treatment benefit, and the characteristics that PROMs should include to best determine patient status and treatment benefit in clinical practice and clinical trials.

The results of this study reveal that frequency of cough and interference of shortness of breath (upon exertion) should be measured daily, given that they do not follow a consistent daily pattern. Severity of physical functioning and ADL limitations should be measured weekly, as some people will not engage in physical activity from one day to the next due to disease. Interference of emotional impacts related to PF-ILD may also vary weekly. Shortness of breath on exertion was considered a key symptom; however, several challenges to its assessment exist, including patients adapting to their symptoms (by restricting their activities) and effects of associated treatments such as supplemental oxygen and physical training.

None of the existing PROMs evaluate the preferred characteristics of the prioritised signs/symptoms and impacts using the proposed recall periods and response scales. Among the reviewed PROMs, the L-PF questionnaire was considered by participants to best capture symptoms and impacts of importance. Direct patient input in its development supports the face validity of the L-PF for assessing symptoms and HRQoL in patients with PF-ILD [16]. In addition, the L-PF has a 24-h recall period for symptoms and 1 week for impacts, and measures most of the prioritised concepts according to its most appropriate characteristic. However, the limited number of fatigue-related items in its impacts module may not be sufficient to support labelling claims on fatigue. Further research is needed to validate the L-PF in patients with PF-ILD, and a shortened version of the L-PF may be more practical for use in clinical trials and clinical practice [10]. The K-BILD is the only other ILD-specific instrument developed for the ILD population. It was previously used to assess the effect of ambulatory oxygen on quality of life in the AmbOx trial, which showed clinically significant effects of an intervention on a PROM for the first time in a randomised controlled trial in ILD [28]. However, K-BILD does not include items to assess the management of cough [29]. Depending on the context of the drug being investigated, the L-PF and K-BILD can be complemented by other PROMs (concept-specific for fatigue or cough).

The panel discussions suggested that maintenance or improvement in symptoms and impacts of PF-ILD would support evidence of treatment benefit. Our intention is not to make a specific recommendation on the type of end-point used in clinical trials (change from baseline, time to event, responder analysis), although discussions highlighted the need for meaningful change thresholds in PROMs for interpreting responder analyses, derived using suitable Patient Global Impression scales [12, 30]. Our results support the concept that assessing patient perspectives are increasingly recognised as important end-points in clinical trials. Future considerations that warrant further discussion include wearable technologies [31] to supplement PROMs by recording response data (*e.g.* physical activity) [32–34]. In designing clinical trials, stratifying patient data based on baseline factors may better qualify influences in the overall burden and patient experience, since HRQoL scores may not consider age and comorbidities [35, 36].

The strengths of this study include the multi-stakeholder involvement and the use of different approaches to prioritise concepts and reach consensus. Consistent with United States Food and Drug Administration guidelines, we identified concepts and instruments for PROM assessment based on literature reviews and expert opinion [12]. There are some limitations to this study. The stakeholders were recruited from the USA and Europe, so the results of this study may not be generalisable to all countries. Another limitation is the small sample size of the stakeholder groups, with relatively low patient input within this consensus panel to drive the prioritisation of concepts for treatment benefit. The systematic literature review included only English-language publications within the past 10 years. However, it is unlikely that the search missed additional symptoms or impacts. The study also focused mainly on pulmonary involvement and did not include other aspects of disease, such as treatment side-effects and extrapulmonary disease manifestations. Although specific PROMs capturing adverse effects of treatments have been developed [37], we did not include this concept in our assessment. Finally, Jeff Swigris, an expert in this field and study participant, is also the developer of the L-PF, so his involvement could conceivably have introduced bias.

Overall, a conceptual model of symptoms and impacts in PF-ILD was developed using a comprehensive multidisciplinary approach that takes into account qualitative research and a literature review. We identified signs/symptoms and impacts found to be most important for measuring treatment effect in PF-ILD, from the perspective of key stakeholders. Of existing PROMs, the L-PF was considered to best capture the signs/symptoms and impacts identified as most important to multiple stakeholders. A combination of PROMs may be most appropriate depending on the study objectives. However, additional patient-centred research is needed to further understand important symptoms and impacts of PF-ILD, including surveys and interviews with patients to prioritise concepts and validate PROMs. Further studies are needed to validate the conceptual model against speciality groups and across different countries to achieve agreement on the concepts to assess treatment benefit.

## Supplementary material

10.1183/23120541.00681-2021.Supp1 **Please note:** supplementary material is not edited by the Editorial Office, and is uploaded as it has been supplied by the author.
Supplementary material 00681-2021.SUPPLEMENT
