# Supplementary material for: Developing a conceptual model of symptoms and impacts in progressive fibrosing interstitial lung disease to evaluate patient-reported outcome measures
Source: ERJ Open Res. 2022 May 3;8(2):00681-2021. doi: 10.1183/23120541.00681-2021 (PMC9062300; doi:10.1183/23120541.00681-2021)
Supplement: Supplementary file 1 [file 00681-2021.SUPPLEMENT.pdf]

Supplementary materials

Developing a conceptual model of symptoms and impacts in progressive fibrosing interstitial lung disease to evaluate patient-reported outcome measures

Authors: Marlies Wijsenbeek,[1] Maria Molina-Molina,[2] Olivier Chassany,[3,4] John Fox,[5] Liam Galvin,[6] Klaus Geissler,[7] Katherine M. Hammitt,[8] Michael Kreuter,[9] Teng Moua,[10] Emily C. O'Brien,[11] Ashley F. Slagle,[12] Anna Krasnow,[13] Matthew Reaney,[14] Michael Baldwin,[15] Natalia Male,[15] Klaus B. Rohr,[15] Jeff Swigris,[16] Katerina Antoniou[17]

Affiliations: [1]Department of Respiratory Medicine, Erasmus University Medical Centre, Rotterdam, The Netherlands; [2]Department of Pneumology, Unit of Interstitial Lung Diseases, University Hospital of Bellvitge, Institute for Biomedical Research (IDIBELL), Barcelona, Spain; [3]Patient-Reported Outcomes Research Unit, Université de Paris, Paris, France; [4]Health Economics Clinical Trial Unit (URC-ECO), Hotel-Dieu Hospital, AP-HP, Paris, France; [5]Foxworthy Healthcare Consulting, Ada, MI, USA; [6]European Idiopathic Pulmonary Fibrosis and Related Disorders Federation, Overijse, Belgium; [7]Patient Support Group Lungenfibrose eV, Essen, Germany; [8]Sjögren's Foundation, Reston, VA, USA; [9]Center for Interstitial and Rare Lung Diseases, Department of Pneumology, Thoraxklinik, University of Heidelberg, Member of the German Center for Lung Research, Heidelberg, Germany; [10]Division of Pulmonary and Critical Care Medicine, Mayo Clinic, Rochester, MN, USA; [11]Duke Clinical Research Institute, Durham, NC, USA; [12]Aspen Consulting, LLC, Steamboat Springs, CO, USA; [13]Patient Centred Solutions, IQVIA, London, UK; [14]Patient Centred Solutions, IQVIA, Reading, UK; [15]Boehringer Ingelheim International GmbH, Ingelheim am Rhein, Germany; [16]National Jewish Health, Denver, CO, USA; [17]Laboratory of Cellular and Molecular Pneumonology, Department of Respiratory Medicine, School of Medicine, University of Crete, Crete, Greece.

26 **Supplementary Table 1** Search strategy in Embase, Medline and Medline in Process

ID	Search	Hits
1	exp Lung Diseases, Interstitial/ 131609	131,609
2	exp Idiopathic Pulmonary Fibrosis/	28,602
3	(ILD or 'ILDs' or 'interstitial lung disease' or 'interstitial lung diseases' or 'idiopathic pulmonary fibrosis' or 'cryptogenic fibrosing alveolitis' or 'fibrocystic pulmonary dysplasia' or 'hamman rich' or 'interstitial pneumonitis').ab,ti.	54,738
4	('progressive fibrosis' or 'progressive fibrosing' or (('progressive' or 'progressing') and ('fibrosis' or 'fibroses' or 'fibrosing'))).ti,ab.	31,812
5	('fast progressing' or 'fast progression' or 'rapid progress' or 'rapid progressing' or 'rapid progression' or 'rapidly progressive' or 'progressive phenotype' or ('progressive' and 'phenotype')).ti,ab.	71,259
6	('pf ild' or 'pf ilds' or 'IPF' or 'pulmonary fibrosis interstitial lung disease' or 'pulmonary fibrosis interstitial lung diseases' or 'progressive fibrosis interstitial lung disease' or 'progressive fibrosis interstitial lung diseases' or 'idiopathic pulmonary fibrosis').ti.	12,298
7	(iNSIP or 'non-specific interstitial pneumonia' or 'Unclassifiable idiopathic interstitial pneumonias' or 'SSc-ILD' or 'RA-ILD' or 'MCTD-ILD' or 'Sjogren syndrome-related ILD' or 'IPAF' or 'interstitial pneumonia with autoimmune features' or 'Chronic hypersensitivity pneumonitis' or 'Sarcoidosis').ab,ti.	57,505
8	(1 or 2 or 3) and (4 or 5)	12,997
9	6 or 7 or 8	82,258
10	exp Quality of Life/	668,202
11	exp Activities of Daily Living/ or exp Patient Reported Outcome	212,332

ID	Search	Hits
	Measures/	
12	('clinical manifestations' or 'clinical characteristics').ab,ti.	300,417
13	('patient reported outcomes' or 'PRO' or 'patient-reported' or 'patient perspective' or 'patient-centricity' or 'patient-centred' or 'patient-centered' or 'patient's perception' or 'patient's perceptions' or 'patient-rated' or 'patientrating').ab,ti.	620,676
14	('signs' or 'symptoms' or 'impacts').ab,ti.	2,870,932
15	('dyspnea' or 'dyspnoea' or 'cough' or 'fatigue' or 'pain').ab,ti.	1,866,194
16	10 or 11 or 12 or 13 or 14 or 15	5,606,461
17	(('experience' or 'experiences' or 'experienced' or 'self report*' or 'self evaluat*' or 'self completed' or 'self administered' or 'self assess*' or 'diary' or 'diaries' or 'qualitative' or 'interview*' or ('concept' and 'elicitation')) and ('patient' or 'patients' or 'participant' or 'subject')).ab,ti.	1,900,603
18	('patient interview' or 'patient interviews' or 'qualitative interview' or 'qualitative interviews' or 'semi-structured interviews' or 'semi-structured interview' or 'survey' or 'conceptual model' or 'focus group').ab,ti.	1,303,240
19	('study' and ('longitudinal' or 'observational' or 'cohort' or 'case series' or 'humanistic')).ab,ti.	1,684,215
20	17 or 18 or 19	4,471,118
21	9 and 16 and 20	2,039
22	limit 21 to yr="2010 -Current"	1,633
23	remove duplicates from 22	1,278

Supplementary Table 2 Search strategy in Cochrane

ID	Search strategy	Hits
#1	"lung diseases, interstitial"[MeSH Terms]	786
#2	"idiopathic pulmonary fibrosis"[MeSH Terms]	285
#3	ILD:ti,ab,kw OR "ILDs":ti,ab,kw OR "interstitial lung disease":ti,ab,kw OR "interstitial lung diseases":ti,ab,kw OR "idiopathic pulmonary fibrosis":ti,ab,kw OR "cryptogenic fibrosing alveolitis":ti,ab,kw OR "fibrocystic pulmonary dysplasia":ti,ab,kw OR "hamman rich":ti,ab,kw OR "interstitial pneumonitis":ti,ab,kw	2,014
#4	"progressive fibrosis":ti,ab,kw OR "progressive fibrosing":ti,ab,kw OR (("progressive":ti,ab,kw OR "progressing":ti,ab,kw) AND ("fibrosis":ti,ab,kw OR "fibroses":ti,ab,kw OR "fibrosing":ti,ab,kw))	615
#5	"fast progressing":ti,ab,kw OR "fast progression":ti,ab,kw OR "rapid progress":ti,ab,kw OR "rapid progressing":ti,ab,kw OR "rapid progression":ti,ab,kw OR "rapidly progressive":ti,ab,kw OR "progressive phenotype":ti,ab,kw OR ("progressive":ti,ab,kw AND "phenotype":ti,ab,kw)	657
#6	"pf ild"[Title] OR "pf ilds"[Title] OR "IPF"[Title]	877
#7	iNSIP:ti,ab,kw OR "non-specific interstitial pneumonia":ti,ab,kw OR "Unclassifiable idiopathic interstitial pneumonias":ti,ab,kw OR "SSc-ILD":ti,ab,kw OR "RA-ILD":ti,ab,kw OR "MCTD-ILD":ti,ab,kw OR "Sjogren syndrome-related ILD":ti,ab,kw OR "IPAF":ti,ab,kw OR "interstitial pneumonia with autoimmune features":ti,ab,kw OR "Chronic hypersensitivity pneumonitis":ti,ab,kw OR "Sarcoidosis":ti,ab,kw	747
#8	(#1 OR #2 OR #3) AND (#4 OR #5)	172
#9	#6 OR #7 OR #8	1,658

ID	Search strategy	Hits
#10	"quality of life"[MeSH terms]	23,061
#11	"patient reported outcome measures"[MeSH Terms]	492
#12	"activities of daily living"[MeSH Terms]	9,063
#13	"patient reported outcomes":ti,ab,kw OR "PRO":ti,ab,kw OR "patient-reported":ti,ab,kw OR "patient perspective":ti,ab,kw OR "patient-centricity":ti,ab,kw OR "patient-centred":ti,ab,kw OR "patient-centered":ti,ab,kw OR "patient's perception":ti,ab,kw OR "patient's perceptions":ti,ab,kw OR "patient-rated":ti,ab,kw OR "patient-rating":ti,ab,kw	30,420
#14	"signs":ti,ab,kw OR "symptoms":ti,ab,kw OR "impacts":ti,ab,kw	164,075
#15	"clinical manifestations":ti,ab,kw OR "clinical characteristics":ti,ab,kw	6,263
#16	"dyspnea":ti,ab,kw or "dyspnoea":ti,ab,kw or "cough":ti,ab,kw or "fatigue":ti,ab,kw or "pain":ti,ab,kw	204,604
#17	#10 OR #11 OR #12 OR #13 OR #14 OR #15 OR #16	368,634
#18	("experience":ti,ab,kw OR "experiences":ti,ab,kw OR "experienced":ti,ab,kw OR "self report*":ti,ab,kw OR "self evaluat*":ti,ab,kw OR "self completed":ti,ab,kw OR "self administered":ti,ab,kw OR "self assess*":ti,ab,kw OR "diary":ti,ab,kw OR "diaries":ti,ab,kw OR "qualitative":ti,ab,kw OR "interview*":ti,ab,kw OR ("concept":ti,ab,kw AND "elicitation":ti,ab,kw)) AND ("patient":ti,ab,kw OR "patients":ti,ab,kw OR "participant":ti,ab,kw OR "subject":ti,ab,kw)	105,049
#19	"patient interview":ti,ab,kw OR "patient interviews":ti,ab,kw OR "qualitative interview":ti,ab,kw OR "qualitative interviews":ti,ab,kw OR "semi-structured interviews":ti,ab,kw OR "semi-structured interview":ti,ab,kw OR "survey":ti,ab,kw OR "conceptual	32,096

ID	Search strategy	Hits
	model":ti,ab,kw OR "focus group":ti,ab,kw	
#20	"study":ti,ab,kw AND ("longitudinal":ti,ab,kw OR "observational":ti,ab,kw OR "cohort":ti,ab,kw OR "case series":ti,ab,kw OR "humanistic":ti,ab,kw)	70,256
#21	("double blind":ti,ab,kw OR "double blinded":ti,ab,kw OR RCT:ti,ab,kw OR Randomi*:ti,ab,kw OR controlled:ti,ab,kw OR controled:ti,ab,kw OR control:ti,ab,kw OR Placebo:ti,ab,kw OR Trial:ti,ab,kw)	314,785
#22	(Study:ti,ab,kw OR studies:ti,ab,kw) AND (open:ti,ab,kw OR open-label:ti,ab,kw OR non-randomised:ti,ab,kw OR nonrandomized: ti,ab,kw OR "cohort":ti,ab,kw)	51,349
#23	#18 OR #19 OR #20 OR #21 OR #22	473,612
#24	#9 AND #17 AND #23	220
#25	#24 last 10 years	189

30 **Supplementary Table 3** Signs, symptoms and impacts relevant in PF-ILD identified by participants in
31 the online survey as pertinent to understanding PF-ILD

**Sign/symptom from the preliminary conceptual model***	**Patient representatives (n=3)**	**Clinicians (n=5)**	**Payer advisors (n=2)**	**Regulatory advisors (n=2)**	**Consensus for priority[#]**
Change in appetite		1 (EU)	0	0	
Chest congestion		0	0	1 (US)	
Chest crackles		0	0	1 (EU)	
Chest discomfort		2 (US)	1 (US)	2 (EU/US)	Yes
Chest pain		0	2 (EU/US)	2 (EU/US)	Yes
Constipation		0	0	1 (EU)	
Cough	1 (EU)	5 (EU/US)	2 (EU/US)	2 (EU/US)	Yes
Difficulty bringing up phlegm/mucous	1 (EU)	0	0	1 (US)	
Dizziness/light headedness	1 (EU)	0	0	0	Yes
Dry mouth		0	0	2 (EU/US)	
Fatigue	1 (EU)	4 (EU/US)	1 (EU)	2 (EU/US)	Yes
Faecal incontinence/diarrhoea	1 (EU)	0	0	1 (EU)	
Fever		0	1 (EU)	0	
Finger/nail clubbing		0	0	2 (EU/US)	
Headaches		0	1 (EU)	0	

Itchy eyes		0	1 (EU)	0	
Loss of appetite		0	0	2 (EU/US)	
Nausea		0	1 (US)	1 (EU)	
Other chest symptoms (chest – tightness, heaviness, pressure)		1 (EU)	2 (EU/US)	2 (EU/US)	Yes
Pain		0	1 (EU)	1 (EU)	
Producing phlegm/mucous		0	0	2 (EU/US)	
Reduced nasal congestion/runny nose	1 (EU)	0	0	0	Yes
Reduced phlegm symptoms	1 (EU)	0	0	0	Yes
Shortness of breath at rest	1 (EU)	3 (EU/US)	1 (US)	2 (EU/US)	Yes
Shortness of breath on exertion	2 (EU)	5 (EU/US)	2 (EU/US)	1 (US)	Yes
Weight loss		0	0	1 (EU)	
Wheezing or whistling sounds		0	0	2 (EU/US)	
**Impact from the preliminary conceptual model***	**Patient representatives (n=3)**	**Clinicians (n=5)**	**Payer advisors (n=2)**	**Regulatory advisors (n=2)**	
Anxiety		1 (US)	1 (US)	1 (EU)	Yes
Ashamed/embarrassed		1 (US)	0	0	

Concern with treatment-related adverse events/side effects		0	0	1 (EU)	
Concerned for family	2 (EU)	0	0	1 (EU)	Yes
Decreased sexual life		0	0	1 (EU)	
Denial about the disease		1 (EU)	0	0	
Depression		1 (US)	1 (US)	1 (EU)	Yes
Difficulty in understanding the implication of disease	1 (EU)	1 (EU)	0	0	Yes
Emotional impact (overall)	1 (EU)	2 (EU/US)	2 (EU/US)	1 (EU)	Yes
Fear		0	0	1 (EU)	
Fear of infection	1 (EU)	1 (EU)		1 (EU)	Yes
Feeling grief		1 (US)	0	0	
Hesitance to engage in advance care planning		0	1 (US)	0	
Impact on ADLs	1 (EU)	3 (EU/US)	2 (EU/US)	2 (EU/US)	Yes
Impact on social activities/travel/holidays		0	0	1 (EU)	
Impact on social life		0	1 (EU)	1 (EU)	
Impaired sleep		0	1 (EU)	2 (EU/US)	
Inconvenience due to		0	0	1 (EU)	

use of oxygen cylinder					
Loss of independence/freedom		3 (EU/US)	0	0	Yes
Low physical activity/loss of functional ability	2 (EU)	3 (EU/US)	0	2 (EU/US)	Yes
Oxygen use having impact on ADLs	1 (EU)	1 (US)	0	1 (EU)	Yes
Panic		0	1 (US)	0	
Stigma/shame associated with oxygen use		1 (EU)	0	0	
Thoughts of death		2 (EU/US)	0	0	Yes
Uncertainty on disease progression	1 (EU)	3 (EU/US)	0	1 (EU)	Yes

The table lists only those signs and symptoms that were selected by at least one stakeholder.

*Preliminary conceptual model developed from the SLR.

#Concepts for inclusion in the first consensus meeting were selected based on the number of stakeholders who indicated concept as important in the online survey and prioritisation by clinical experts and patient representatives.

ADL, activities of daily living; EU, European Union; PF-ILD, progressive fibrosing interstitial lung disease; reps, representatives; US, United States.

Supplementary Figure 1 PRISMA flow diagram with the identified studies

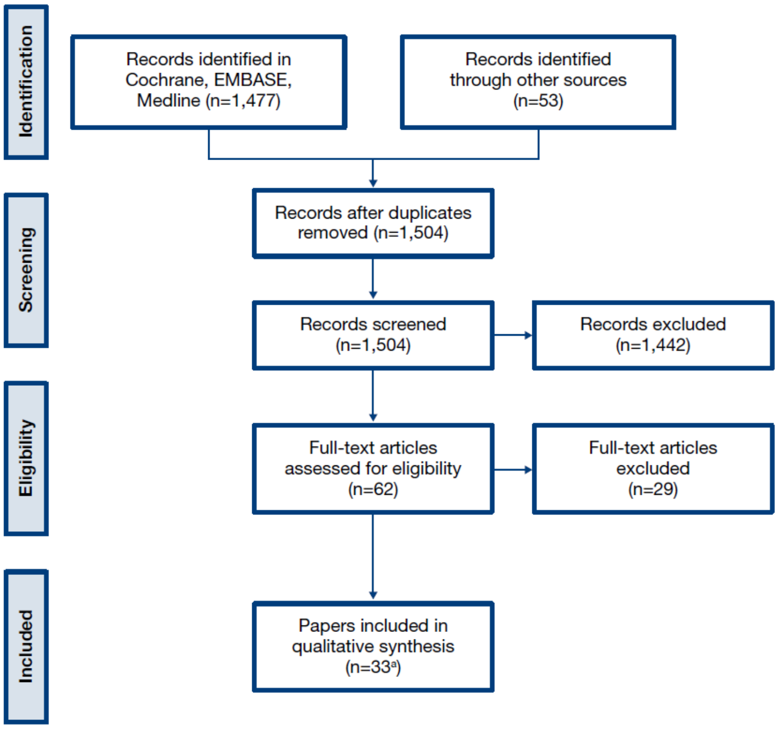

[a]Including 49 entries in 10 patient blogs. Each blog (not the entry) was considered as a hit in the PRISMA flow.
